# Chestnut Wood Residues, with and Without Tannins, as a Potential Feedstock for PHA Bioplastic Production

**DOI:** 10.3390/polym18101206

**Published:** 2026-05-15

**Authors:** Jasmina Jusic, Alessandra Filieri, Silvia Crognale, Matteo Manni, Swati Tamantini, Vittorio Vinciguerra, Alessandro Cardarelli, Marco Barbanera, Dennis Jones, Dominik Matt, Manuela Romagnoli

**Affiliations:** 1 Department of Innovation in Biological, Agro-Food and Forest Systems (DIBAF), University of Tuscia, 01100 Viterbo, Italy; alessandra.filieri@unitus.it (A.F.); matteo.manni@unitus.it (M.M.); swati.tamantini@unitus.it (S.T.); vincigue@unitus.it (V.V.); 2 Fraunhofer Italia Research Innovation Engineering Center, 39100 Bolzano, Italy; dominik.matt@fraunhofer.it; 3 Department of Economics Engineering Society and Business Organization (DEIM), University of Tuscia, 01100 Viterbo, Italy; a.cardarelli@unitus.it (A.C.); m.barbanera@unitus.it (M.B.); 4 Wood Science and Engineering, Luleå University of Technology, 93187 Skellefteå, Sweden; dennis.jones@ltu.se; 5 Faculty of Science and Technology, Free University of Bozen-Bolzano, Piazza Università 4, 39100 Bolzano, Italy

**Keywords:** biodegradable polymers, polyhydroxyalkanoates (PHA), exhausted chestnut wood, tannins, *Cupriavidus necator*, hardwood

## Abstract

The valorisation of lignocellulosic residues into bio-based feedstocks is a key strategy for advancing circular bioeconomy models. In this study, chestnut wood residues, including virgin wood (VW) and detannized wood (DT) from the tannin industry, were evaluated as substrates for polyhydroxyalkanoate (PHA) production using *Cupriavidus necator*. Biomass was subjected to thermo-acid hydrolysis followed by ion-exchange detoxification, yielding hydrolysates rich in organic acids (levulinic, acetic, and formic acids) and residual inhibitory compounds. Both substrates supported microbial growth and PHA accumulation, although clear differences in performance were observed. The maximum biomass concentration reached 1.26 ± 0.01 g L^−1^ in VW hydrolysate and 0.40 ± 0.03 g L^−1^ in DT hydrolysate. PHA production was higher in VW hydrolysate, reaching 68.51 mg L^−1^ with 5.44% (*w*/*w*) accumulation, while DT hydrolysate yielded 0.21 mg L^−1^ with 6.01% (*w*/*w*). The reduced biomass formation in DT hydrolysate was associated with the greater persistence of inhibitory compounds generated during thermo-acid treatment. Although the obtained PHA yields are lower than those reported for optimized lignocellulosic systems, this study demonstrates for the first time the feasibility of producing PHA from chestnut wood residues, including industrial detannized byproducts, without nutrient supplementation. These findings highlight the potential of tannin-industry waste streams as alternative feedstocks for biopolymer production, while indicating that optimization of hydrolysis conditions, detoxification efficiency, and fermentation strategy is required to improve process performance.

## 1. Introduction

Global plastic production has increased dramatically over the past decades, with an average annual growth rate of around 9%, and it is projected to reach 540 million metric tons by 2040 [1,2,3]. Growing environmental concerns associated with plastic waste have driven the development of biopolymers capable of degrading in various environments [4,5]. Among bioplastics, polylactic acid (PLA) is produced from renewable feedstocks such as starch-rich crops (e.g., corn, sugarcane, and potatoes). Similarly, bio-based polyurethanes are derived from vegetable oils, including soybean, castor, and rapeseed oil. Lignocellulose, the most abundant raw material on Earth, consists of two linear polymers—cellulose and hemicellulose, along with a nonlinear lignin polymer [6]. It represents a rich source of carbon and chemical energy, making its recycling essential for maintaining the global carbon cycle [7]. Despite its abundance, the use of lignocellulosic biomass from forestry or wood industry as a feedstock for bioplastic production remains largely underexplored. Within the expanding spectrum of biobased polymers, polyhydroxyalkanoates (PHAs) represent a unique class of biodegradable polymers, synthesized intracellularly by various microorganisms as carbon and energy reserves when carbon sources are in excess and at least one other nutrient is limiting [8]. Unlike traditional plastics, which can take between 100 and 1000 years to degrade, PHA-based bioplastics can degrade into water (H_2_O) and carbon dioxide (CO_2_) within 20 to 45 days under suitable conditions, including adequate humidity, oxygen availability and microbial activity [9,10]. Among hardwoods, particular attention is given to chestnut wood (*Castanea sativa* Mill), a tannin-rich species that has not yet been explored as a potential feedstock for PHA production. In Italy, chestnut forests cover approximately 780,000–800,000 hectares, accounting for about 7.5–9.2% of the national forest area and roughly 2.6% of the total land area, according to the Ministry of Agriculture Policies [11]. In addition to chestnut, other tannin-rich species such as oak (*Quercus* spp.), quebracho (*Schinopsis* spp.), and mimosa (*Acacia* spp.) are widely used in tannin extraction industries and may represent alternative feedstocks for similar valorization pathways. However, their potential for PHA production remains largely unexplored. Tannin extraction industries generate considerable quantities of exhausted wood residues that retain valuable chemical properties [12,13,14] and, together with virgin wood residues, represent promising feedstocks for bioplastic production. While previous studies have demonstrated PHA production from lignocellulosic substrates, particularly from conifer-derived hydrolysates, research on hardwood residues, and especially on industrial byproducts from tannin extraction processes, remains limited. Moreover, no studies to date have compared the performance of virgin and detannized hardwood biomass under identical processing and fermentation conditions. This gap represents a significant challenge in the development of lignocellulosic biorefineries. Based on these considerations, this study aims to evaluate the suitability of chestnut wood-derived lignocellulosic residues for PHA production by adapting and assessing protocols previously developed for coniferous biomass. Chestnut wood residues were subjected to thermo-acid hydrolysis followed by ion-exchange detoxification, and the resulting hydrolysates were directly used—without nutrient supplementation—for microbial cultivation. By comparing untreated and industrially processed biomass, this study introduces chestnut wood residues as a novel feedstock for PHA production and provides new insights into the role of tannin extraction in lignocellulosic bioconversion processes within a circular bioeconomy framework.

## 2. Materials and Methods

**Figure 1:**
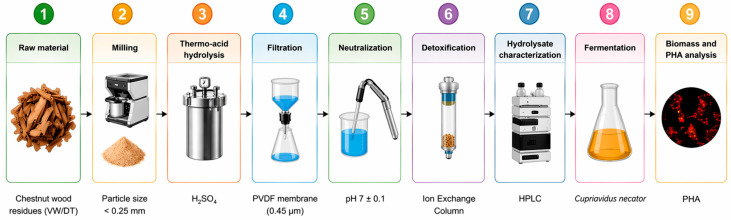
Experimental workflow for PHA production from chestnut wood residues (VW/DT), including biomass preparation, thermo-acid hydrolysis, filtration, neutralization, ion-exchange detoxification, hydrolysate characterization, microbial cultivation with *Cupriavidus necator*, and PHA analysis.

The overall experimental workflow is summarized in Figure 1.

### 2.1. Microorganism

*Cupriavidus necator* (LMG 1209; formerly *Ralstonia eutropha*) was used in this study as a model microorganism due to its metabolic versatility and well-established ability to synthesize PHA from diverse carbon substrates, including lignocellulosic-derived compounds [15,16,17]. The strain was provided by the Belgian Coordinated Collections of Microorganisms (BCCM) and maintained at the Laboratory of General and Applied Microbiology, University of Tuscia. The microorganism was preserved on agar slants with the following composition (g L^−1^): beef extract 1, yeast extract 2, peptone 5, NaCl 5, and agar 15, adjusted to pH 7.4.

### 2.2. Wood Biomass

Chestnut wood residues (VW/DT) were prepared as shown in Steps 1–2 of Figure 1. Chestnut wood residues were provided by the Nuova Rivart tannin industry (Radicofani, SI, Italy) and consisted of two different batches of wood chips. The first batch was virgin wood waste (VW), characterized chemically prior to any treatments. The second batch was a detannized wood waste (DT), obtained after industrial tannin extraction. The physicochemical properties of both VW and DT have been previously reported [13]. Virgin wood exhibited a higher extractives content (7.17%), while detannized wood retained a significant fraction of extractives (4.0%), despite industrial processing (see Table 1). Wood chips were air-dried for several weeks, reaching a moisture content of approximately 8.3% for both VW and DT. Ash content was approximately 0.6% for VW and 1.2% for DT, as previously reported in Jusic et al. [13]. The dried material was milled using an Ika M10.1 cutting mill (IKA^®^-Werke GmbH & Co. KG, Staufen, Germany) and sieved through a 35-mesh screen, yielding particles of approximately 0.25 mm. No detailed particle size distribution analysis was performed.

### 2.3. Hydrolysis Reaction

Thermo-acid hydrolysis was performed as illustrated in Step 3 of Figure 1. Biomass hydrolysis was carried out in a Parr 4560 mini-batch reactor (Parr Instrument Company, Moline, IL, USA). The reactor was loaded with 10 wt% of milled chestnut wood flour (particle size < 0.25 mm), suspended in a 5% (*v*/*v*) sulfuric acid solution. Prior to the reaction, the system was purged with nitrogen gas to eliminate residual air. Agitation was maintained at a stirring rate of 200 rpm. The reactor was heated at a rate of 2.3 °C/min to 200 °C, and held at this temperature for 90 min, after which it was allowed to cool passively to room temperature.

The resulting solid–liquid slurry was separated by vacuum filtration using a 0.22 µm polyvinylidene fluoride (PVDF) membrane filter (Merck KGaA, Darmstadt, Germany). The filtrate, including wash water, was combined and adjusted to a final volume of 500 mL with water. This solution, referred to as the crude hydrolysate, was stored at 4 °C prior to further characterization. The selected hydrolysis conditions were intentionally severe to promote organic acids formation rather than maximize sugar recovery, following previously reported protocols for lignocellulosic biomass conversion [16].

### 2.4. Post-Reaction Detoxification

Following hydrolysis, the samples were filtered, neutralized, and detoxified through ion exchange treatment (Steps 4–6, Figure 1). Ion exchange column (IEC) detoxification was applied to remove inhibitory ionic compounds via reversible exchange with functional groups on a solid resin matrix. This method was selected based on its demonstrated effectiveness compared to alternatives such as activated carbon and overliming for supporting *Cupriavidus necator* growth [16]. Amberlyst 36 H^+^, a strong acid cation-exchange resin with sulfonic acid functional groups, was used. The resin removes inhibitory cationic species and organic acid–related impurities known to impair microbial metabolism. As reported by Mohan et al. [16] IEC treatment significantly reduced fermentation inhibitors while preserving fermentable substrates, enabling approximately 70% relative growth on levulinic acid compared to the non-detoxified hydrolysate. The effectiveness of IEC is attributed to its high selectivity, minimal sugar loss, and ability to operate under mild conditions compared to alternative detoxification strategies. A 100 mL burette was packed with cotton and washed sand to form the base, then filled with water and resin, ensuring no air bubbles were present. A total of 25 g of Amberlyst resin was added, forming a packed bed (1.5 cm diameter × 20 cm length) with a void volume of 15 mL. Prior to passing the crude hydrolysate solution through the bed, the resin was washed sequentially with 300 mL of distilled water, followed by 200 mL of 5 wt% sulfuric acid. The first 15 mL of effluent was discarded to minimize dilution effects. The hydrolysate was neutralized with NaOH to pH 7.0 ± 0.1 before detoxification and subsequent microbial cultivation.

### 2.5. Shake Flask Cultures

The microorganism (*Cupriavidus necator*) was cultivated in 1000 mL baffled flasks containing 250 mL of either virgin wood hydrolysate (VWH) or detannized wood hydrolysate (DTH), used directly as culture media (Figure 1, step 8). Flasks were inoculated with 10% (*v*/*v*) of a pre-inoculum grown on a complex medium containing (g L^−1^): beef extract 1, yeast extract 2, peptone 5, and NaCl 5. Cultures were incubated at 28 °C and agitated at 150 rpm in an orbital shaker incubator (Innova^®^ 40/40R) for 7 days. All experiments were performed in duplicate. Samples were collected every 24 h under sterile conditions to monitor growth kinetics. Optical density (OD_600_) was measured using a UV-1800 SHIMADZU spectrophotometer, providing a relative estimation of cell density. Cell dry weight was determined by centrifugation at 9000 rpm for 10 min. The resulting cell pellets were washed with distilled water to remove residual impurities, stored at −20 °C and lyophilized for 24 h. The lyophilized biomass was weighed and used for PHA extraction. The supernatant solution was analyzed with HPLC.

### 2.6. Chemical Analysis

#### 2.6.1. Hydrolysate Characterization

Hydrolysates and culture supernatants were analyzed using an Agilent 1100 HPLC system equipped with a column thermostat and a diode array detector using Agilent Chemstation (version B.03.02) for operation (Agilent Technologies Inc., Santa Clara, CA, USA). An organic acid column (Sepachrom OA-1000; 9 µm; 300 × 7.8 mm, SepaChrom S.r.l., Rho, Milan, Italy) and guard column (Sepachrom IOA-1000 20 × 4 mm Guard cartridge in a 20 × 40 mm holder, SepaChrom S.r.l., Rho, Milan, Italy) were maintained at 65 °C. A diode array detector was set at 210 and 280 nm to detect the elutants. A 5-μL injection was eluted at 0.450 mL/min with 5 mM sulfuric acid prepared in ultra-pure water. A 5-point calibration curve was prepared for acetic, formic and levulinic acid, hydroxymethylfurfural (HMF) and furfural standards, using analytical-grade chemicals purchased from Carlo Erba Reagents S.r.l. (Milan, Italy). The method is consistent with previously reported protocols for lignocellulosic hydrolysate analysis [16,18]. Total phenolic content (including tannins and low molecular weight phenols) was determined using the Folin–Ciocalteu assay [19], expressed as gallic acid equivalents (GAE). Briefly, 50 μL of the hydrolysate was diluted in 1.2 mL of distilled water, followed by 1.25 mL of the Folin–Ciocalteu reagent. After 6 min of incubation, 12.5 mL of 7% (*w*/*v*) Na_2_CO_3_ solution was added. The mixture was incubated at room temperature for 90 min, and absorbance was measured at 760 nm. HPLC analysis was performed at time 0 and subsequently repeated in duplicate on samples collected after 48 h (time 1), 120 h (time 2), and 168 h (time 3) of cell growth to monitor the evolution of organic acids and other soluble metabolites, as shown in Figure 1 (step 7).

#### 2.6.2. PHA Determination

PHA was extracted from lyophilized biomass using chloroform extraction [20,21]. The dried cells were subjected to methanolysis to convert PHA monomers into their corresponding methyl esters, for gas chromatography–mass spectrometry (GC–MS) analysis. For methanolysis, 50 mg of lyophilized biomass was mixed with 2 mL of chloroform, 1.7 mL of methanol and 0.3 mL of 98% sulfuric acid as a catalyst. The reaction mixture was incubated at 100 °C for 4 h in a thermostated water bath shaker. After cooling to room temperature, 1 mL of distilled water was added to induce phase separation.

The organic phase containing the methyl esters of PHA monomers was analyzed by GC-MS. Benzoic acid was added as an internal standard, prior to methanolysis, and commercial poly(3-hydroxybutyrate) (PHB) (Sigma-Aldrich, St. Louis, MO, USA) was used as an external standard for calibration. Analysis was performed using a GC-MS system (Konik 5000C Sant Cugat del Vallés, Barcelona, Spain) that consisted of a gas chromatograph HRGC 5000C coupled to a Q12 MS mass spectrometer. The capillary column was a Restek Rxi-5MS silica column (30 m × 0.25 mm, 0.25 μm) operating with helium carrier gas at 1.2 mL/min. The injection port was set at 250 °C and operated in split mode (1:50). The oven program ranged from 40 °C to 200 °C at a rate of 10 °C/min. The MS interface was set at 260 °C, while the ion source, operating in electron ionization mode at 70 eV, was set to 180 °C. The quadrupole operated in full scan mode from 41 to 450 m/z. Intracellular PHA accumulation was preliminarily assessed using Nile red fluorescence staining as a qualitative indicator of polymer accumulation (Figure 1, step 9) [22].

### 2.7. Statistical Analysis

Statistical analysis was performed to compare virgin wood (VW) and detannized wood (DT) conditions. A two-tailed Student’s t-test was applied to the main experimental outputs (OD_600_, biomass concentration, and PHA accumulation) at the final cultivation time point. Statistical significance was evaluated at a confidence level of 95% (*p* < 0.05). Results are reported as mean ± standard deviation. Student’s t-test was selected as a commonly used method for preliminary comparative analyses between two experimental groups in microbial and bioprocess studies [23]. Experiments were conducted in duplicate (*n* = 2) due to the exploratory nature of this preliminary feasibility study and the resource-intensive experimental workflow involving thermo-acid hydrolysis, detoxification, and multi-step analytical characterization. The primary objective was to identify general trends and assess the feasibility of utilizing chestnut wood hydrolysates as fermentation substrates rather than to perform a fully optimized statistical evaluation. Therefore, statistical results should be interpreted with caution. Statistical analysis was performed using Microsoft Excel 365 (Microsoft Corp., Redmond, WA, USA).

## 3. Results and Discussion

### 3.1. Biomass Composition

The chemical composition of the lignocellulosic biomass (wood chips) was previously characterized by Jusic et al. [13] and is summarized in Table 1.

Virgin wood (VW) exhibited an extractives content of 7.117%, while cellulose, lignin, and hemicellulose contents were estimated at 36.66%, 31.89%, and 13.9%, respectively. Hemicelluloses consisted predominantly of xylose (96.94%), with a minor contribution from galactose (3.07%). In detannized wood (DT), the extractives (tannin) content decreased to 4.02%, indicating partial removal during industrial processing. Cellulose, lignin, and hemicellulose contents were 37.78%, 29.38%, and 13.40%, respectively. The hemicellulosic fraction consisted mainly of xylose (93.69%) and galactose (5.31%). Unidentified components in both samples were attributed to residual extractives, acetals, pectins, ash (inorganic residue after combustion), and products of sugar degradation. The tannin extraction process had only a minor impact on the relative proportions of the main cell wall components (cellulose and lignin), except for a slight reduction in the soluble lignin fraction. The persistence of tannins, albeit at reduced levels, confirmed that industrial detannization does not completely eliminate phenolic compounds from the biomass, consistent with previous findings [12]. The sum of the compositional fractions slightly exceeds 100%, likely due to cumulative analytical uncertainties and partial overlap between fractions during biomass characterization.

### 3.2. Hydrolysis Yield and Hydrolysate Composition

In this study, hydrolysis yield was estimated based on the percentage of residual solid biomass (cake) recovered after the thermo-acid treatment. The recovered solid fractions were 38.88% for detannized wood (DT) and 38.84% for virgin wood (VW), indicating comparable solid recovery efficiency. The chemical composition of the resulting hydrolysates after thermo-acid treatment is presented in Table 2. Direct comparison of inhibitor profiles from wood hydrolysates is challenging, as their formation strongly depends on the pretreatment conditions and detoxification strategies. Moreover, studies specifically addressing chestnut wood hydrolysis remain scarce.

**Figure 2:**
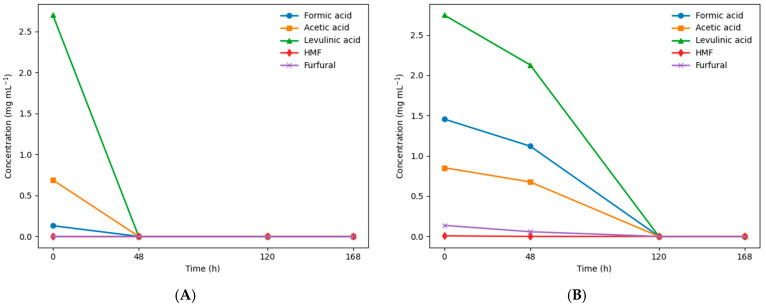
Temporal evolution of the main organic acids detected during cultivation in (**A**) virgin wood hydrolysate (VWH) and (**B**) detannized wood hydrolysate (DTH). Concentration profiles of levulinic, formic, and acetic acids were monitored throughout the fermentation process to evaluate substrate utilization and potential inhibitory effects on microbial growth and PHA accumulation.

Dilute sulfuric acid disrupts the recalcitrant structure of lignocellulose by hydrolyzing hemicellulosic polysaccharides and partially solubilizing amorphous cellulose, releasing fermentable sugars into the liquid phase. However, under the severe conditions used in this study, the sugars such as glucose, xylose and galactose, arabinose and mannose were not detected in the hydrolysate), and this result is consistent with previous observations [16]. The hydrolysate composition was instead rich in degradation products, including organic acids, furans, and phenolic compounds, which are known microbial inhibitors that can reduce fermentation efficiency [24]. In the liquid fraction, levulinic acid (LA), formic acid (FA), and acetic acid (AA) were detected as major components. Levulinic acid formation from C6 carbohydrates (glucose and, in this case, galactose) follows a well-established reaction pathway: acid hydrolysis releases C6 monomers, which are dehydrated to form 5-hydroxymethylfurfural (HMF), a key intermediate commonly detected in wood-derived hydrolysates [16]. HMF is subsequently rehydrated under acidic conditions to yield equimolar amounts of levulinic acid and formic acid. Phenolic content, expressed as gallic acid equivalents (GAE), was substantial in both hydrolysates. Virgin wood hydrolysate (VWH) contained 284.1 mg L^−1^, while detannized hydrolysate (DTH) showed a lower value (214.85 mg L^−1^), reflecting prior extraction of hydrolysable tannins, which are phenolic in nature. Phenolic compounds likely originate from both residual tannins and partial acid-catalyzed lignin degradation. Additionally, the use of sulfuric acid followed by neutralization with NaOH may lead to elevated salt concentrations, potentially inhibiting microbial growth through osmotic stress. Formic and acetic acids contribute to microbial metabolism as carbon sources. Acetic acid is mainly produced from the cleavage of acetyl groups in hemicellulose, while levulinic acid originates from cellulose degradation. However, at elevated concentrations, these compounds may exert inhibitory effects [16]. In VWH, complete conversion of C6 sugars (glucose and galactose) into levulinic acid via HMF was observed, with no detectable accumulation of intermediate compounds. In contrast, residual traces of HMF and furfural were detected in detannized wood (DT), suggesting that conversion of C6 sugars was not fully complete. Although the concentrations of HMF and furfural were low, these lignocellulosic degradation products are known microbial inhibitors and may negatively affect cell growth if not adequately detoxified [25,26,27,28]. In the present study, their concentration remained within manageable levels. The temporal evolution of organic acids in both hydrolysates is presented in Figure 2A,B, highlighting the differences between VW and DT substrates during cultivation.

Compared with other studies on lignocellulosic hydrolysates, the inhibitor levels detected in the present work were relatively moderate. Kucera et al. [24] reported significantly higher concentrations of furans and phenolic compounds in acid-pretreated biomass, which were associated with marked reductions in microbial growth and fermentation efficiency. Similarly, Mohan et al. [16] observed that levulinic acid and related degradation products accumulated at inhibitory levels following thermo-acid hydrolysis, requiring detoxification to restore fermentability. In contrast, the concentrations detected in the present study (Figure 2A,B) were comparatively moderate and decreased over time, suggesting effective microbial utilization. Direct comparison of inhibitor profiles from wood hydrolysates is challenging, as their formation strongly depends on the pretreatment conditions and detoxification strategies employed [29]. Studies specifically addressing chestnut wood hydrolysis are limited. A partial comparison can be drawn with the work of Alhafiz et al. [30], who investigated the cultivation of *Cupriavidus necator* strains on enzymatically hydrolyzed lignocellulosic beech feedstock; however, differences in pretreatment limit direct comparison. As shown in Figure 2A, VWH exhibited the highest concentrations of formic, acetic, and levulinic acids at time 0, followed by a marked reduction after 48 h, indicating their consumption or transformation during microbial growth. Although a complete carbon conversion efficiency was not determined, the observed decrease in organic acid concentrations, together with biomass formation, indicates partial utilization of the available carbon sources. DTH initially exhibited higher concentrations of organic acids and residual inhibitory compounds, including HMF. Organic acids and inhibitory compounds persisted for a longer period, with near-complete consumption occurring only after approximately 120 h. The delayed reduction of organic acids observed in DTH compared with VWH likely reflects differences in hydrolysate accessibility and inhibitor composition induced by the detannization process. Partial removal of tannins may increase the exposure of cellulose and hemicellulose fractions during thermo-acid treatment, promoting deeper polysaccharide degradation and enhanced formation of secondary degradation products such as HMF, furfural, and low-molecular-weight organic acids. Similar effects of lignocellulosic pretreatment severity on inhibitor formation have been widely reported in acid-pretreated biomass hydrolysates [12,31,32,33,34]. Undissociated organic acids can diffuse across the cell membrane and dissociate in the near-neutral cytosolic environment, leading to intracellular acidification and metabolic stress. Despite these inhibitory effects, the inhibitor concentrations in both hydrolysates remained within ranges that still allowed microbial growth and PHA accumulation. This suggests that the applied hydrolysis conditions resulted in a comparatively moderate inhibitor profile compatible with the metabolic tolerance of *Cupriavidus necator*.

### 3.3. Microbial Growth of C. necator on Chestnut Wood Hydrolysates

**Figure 3:**
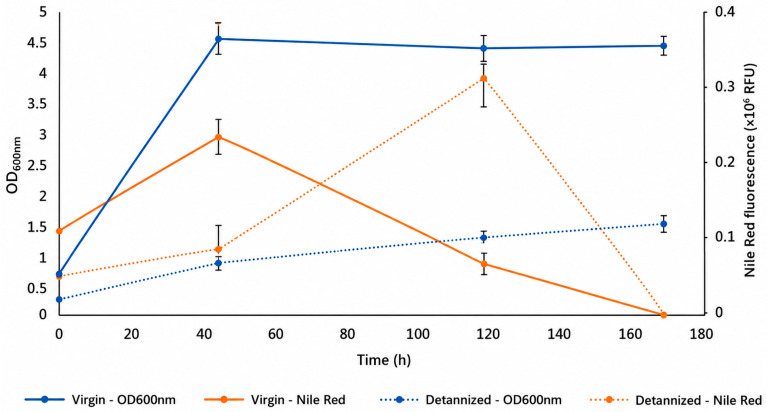
Overlapping profiles of microbial growth (OD_600_, left axis) and intracellular PHA accumulation (Nile Red fluorescence, ×10^7^ RFU, right axis) during 168 h of cultivation of *Cupriavidus necator* in virgin wood (VW) and detannized wood (DT) hydrolysates.

The soil bacterium *C. necator* is a promising microorganism for biorefinery applications, as it grows heterotrophically and lithotrophically, and it can convert excess carbon sources into polyhydroxyalkanoate (mainly poly-3-hydroxybutyrate, PHB) [34]. In this work, the strain was cultivated directly on chestnut wood hydrolysates, without dilution or nutrient supplementation, on both virgin wood hydrolysate and detannized hydrolysate. *Cupriavidus necator* was able to grow on both substrates, even with limited biomass production. Microbial biomass increased progressively up to 120 h of cultivation. The temporal profiles of microbial growth (OD_600_) in both hydrolysates are shown in Figure 3, together with the corresponding intracellular PHA accumulation measured by Nile Red fluorescence. Overall, microbial biomass formation was markedly lower in DTH, compared to VWH. The difference may be attributed to the higher concentration and persistence of inhibitory compounds during thermo-acid treatment.

Tannin removal may enhance biomass accessibility, promoting deeper degradation and the generation of organic acids, HMF, and furfural, which were detected in DTH (Table 2). These compounds, together with salts formed during neutralization, can induce intracellular acidification and osmotic stress, thereby reducing microbial growth efficiency [35]. This trend may be attributed to different concentrations of inhibitory compounds in two hydrolysates (Table 2). These differences may be induced by the detannization process. Removal of tannins from the original feedstock increased the exposure of lignocellulosic cell wall components to chemical fractionation during thermo-acid treatment [36], potentially enhancing the formation of degradation products such as organic acids, HMF, and furfural. The higher concentration of these inhibitory compounds in DTH may therefore have contributed to reduced bacterial growth. However, *Cupriavidus necator* growth continued even in the presence of inhibitors, indicating that its microbial strains were able to tolerate these inhibitors, at least within the applied detoxification strategy. Nile red fluorescence provided a semi-quantitative indication of intracellular PHA accumulation. Maximum PHA accumulation occurred at 48 h in VWH, whereas in DTH the peak was delayed to 120 h, consistent with the slower growth profile.

### 3.4. PHA Production

**Figure 4:**
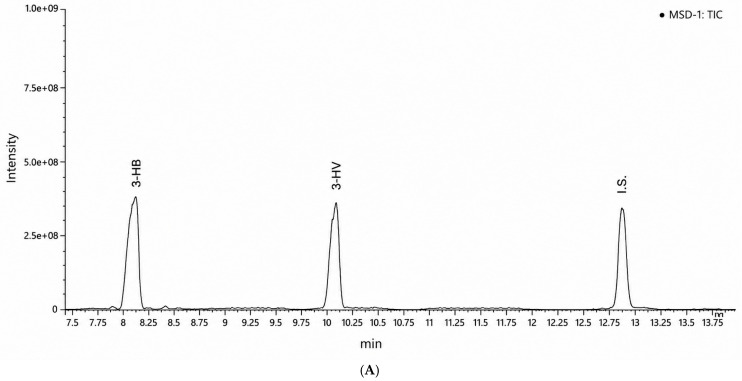

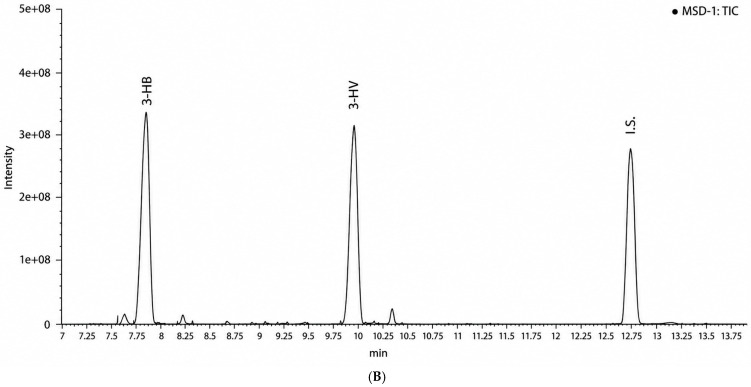
Representative GC–MS chromatograms of biomass samples obtained from *Cupriavidus necator* cultivated on virgin wood hydrolysate (VWH) after (**A**) 24 h and (**B**) 48 h of cultivation. Peaks corresponding to 3-hydroxybutanoic acid methyl ester (3-HB), 3-hydroxypentanoic acid methyl ester (3-HV), and the internal standard (I.S.) are indicated.

PHA production was evaluated by GC-MS analysis of biomass samples collected at 24 h intervals. Representative GC–MS chromatograms of biomass samples collected after 24 h and 48 h of cultivation are shown in Figure 4A,B. The methyl ester of 3-hydroxybutanoic acid (3-HB), corresponding to PHB monomeric units, was detected at a retention time of 8.35 min, while the internal standard, methyl ester of benzoic acid, appeared at 12.83 min, confirming intracellular PHA accumulation. In addition, a second prominent peak corresponding to 3-hydroxypentanoic acid methyl ester (3-HV) was detected in both chromatograms.

As summarized in Table 3, the relative proportions of the 3-HB and 3-HV signals remained approximately constant between 24 h and 48 h cultivation, suggesting the possible formation of a PHBV copolymer containing both hydroxybutyrate and hydroxyvalerate units. Incorporation of hydroxyvalerate monomers is known to improve polymer flexibility and mechanical properties compared with PHB homopolymers [21]. However, additional compositional characterization would be required to confirm copolymer architecture.

After 24 h, PHA content reached 3.1% (*w*/*w*), corresponding to 0.04 g L^−1^. At 48 h, intracellular accumulation increased to 5.5%, corresponding to 0.07 g L^−1^. In DTH, a similar trend was observed, with maximum PHA accumulation occurring at 120 h, coinciding with peak biomass concentration. Monitoring biomass and PHA accumulation enables evaluation of microbial adaptation to different carbon sources and the impact of inhibitory effects of lignocellulosic degradation products [37]. Correlating biomass productivity with PHA yield provides insight into fermentation efficiency and carbon flux distribution [38,39].

PHA production was correlated with biomass formation (Table 4).

In VWH, a final biomass concentration of 1.26 ± 0.01 g L^−1^ resulted in 68.51 mg L^−1^ of PHA, corresponding to a percentage of accumulation of 5.44% after 48 h of cultivation. In DTH, biomass accumulation was significantly lower (0.40 ± 0.03 g L^−1^); however, PHA production was still detected (0.21 mg L^−1^), yielding a Y_P/X_ value of 6.01% after 120 h. Although the absolute PHA concentration in DTH was markedly lower than in VWH, the comparable Y_P/X_ ratio suggests that, under limited growth conditions, a similar fraction of biomass was directed toward polymer accumulation. Preliminary statistical comparison using a two-tailed Student’s t-test did not indicate statistically significant differences between VWH and DTH conditions at the evaluated time points (*p* > 0.05). However, these results should be interpreted cautiously due to the limited replication (n = 2), which reduces statistical power despite the consistent experimental trends observed across measurements. PHA production from lignocellulosic hydrolysates using *C. necator* typically reaches concentrations in the range of approximately 1–4.5 g L^−1^, with intracellular accumulation commonly between 30 and 50% (*w*/*w*), depending on substrate type and process conditions [3]. In comparison, the PHA concentrations obtained in this study (68.51 mg L^−1^ in VWH and 0.21 mg L^−1^ in DTH) and intracellular accumulation (~5–6% *w*/*w*) are significantly lower.

The comparison between VWH and DTH underscores the influence of biomass pretreatment on microbial productivity. Differences in hydrolysate composition and inhibitor profiles likely affected the metabolic performance of *Cupriavidus necator.* The higher biomass and PHA production observed in VWH suggest improved availability of fermentable substrates and/or a lower inhibitory burden compared to DTH, which did not adequately sustain either growth or polymer accumulation. Overall, these findings highlight the importance of hydrolysate composition and pretreatment strategy in optimizing microbial biopolymer production. From a biorefinery and circular bioeconomy perspective, untreated chestnut wood appears to represent a more suitable feedstock for PHA production under the tested conditions. Nevertheless, the relatively low PHA yields obtained in both VWH and DTH indicate that further optimization, particularly through improved detoxification strategies and adjustment of hydrolysis conditions, is necessary to enhance process efficiency. In addition, the potential of DTH as a PHA feedstock could be improved through optimization of treatment conditions.

## 4. Conclusions

This study demonstrates the feasibility of utilizing chestnut wood residues, including both virgin and detannized materials, as feedstocks for polyhydroxyalkanoate (PHA) production using *Cupriavidus necator*. Thermo-acid hydrolysis generated hydrolysates rich in organic acids and inhibitory compounds rather than fermentable sugars, yet microbial growth and PHA accumulation were still achieved without external nutrient supplementation or hydrolysate dilution. Virgin wood hydrolysate supported higher biomass formation and PHA production compared with detannized hydrolysate, highlighting the influence of pretreatment-derived inhibitor profiles on microbial performance. The relatively low polymer accumulation observed in this study is likely associated with the absence of nitrogen limitation, the presence of inhibitory compounds, and non-optimized cultivation conditions. Despite the modest PHA yields obtained, the results provide valuable insight into the valorization of chestnut wood residues and tannin-industry byproducts within a circular bioeconomy framework. In addition, GC–MS analysis suggested the possible incorporation of hydroxyvalerate units within the produced polymer, indicating potential formation of structurally diverse PHAs. Future work should focus on optimization of hydrolysis severity, detoxification efficiency, and fermentation strategies, including nutrient limitation and fed-batch cultivation, to improve microbial productivity and polymer accumulation. Further kinetic and carbon-balance analyses will also be important to better understand metabolic flux distribution and support future process scale-up.

## Figures and Tables

**Table 1:** Chemical composition of virgin wood (VW) and detannized wood (DT) chestnut biomass used in this study, including extractives (tannins), hemicellulose-derived sugars, cellulose, lignin, and non-identified components. Values are expressed as a percentage of dry biomass composition and are adapted from [13].

Sample	Extractives (Tannin) %	Hemicellulose (%)	Xylose (%)	Galactose (%)	Cellulose (%)	Lignin (%)	Not Identified Components (%)
VW	7.11	13.90	96.94	3.07	36.66	31.89	17.56
DT	4.02	13.40	93.69	5.31	37.78	29.38	19.38

**Table 2:** Chemical composition of virgin wood hydrolysate (VWH) and detannized wood hydrolysate (DTH) after thermo-acid hydrolysis, neutralization, and ion-exchange detoxification, prior to microbial cultivation. The table reports the concentrations of the main organic acids, degradation products, and total phenolic compounds potentially affecting microbial growth and PHA production.

Concentration mg mL^−1^	VWH	DTH
**Formic acid**	0.130	1.455
**Acetic acid**	0.688	0.852
**Levulinic acid**	2.699	2.746
**HMF**	-	0.007
**Furfural**	-	0.137
**Total Phenolic content (mg L^−1^ gallic** **acid equivalents)**	284.1 ± 2.26	214.855 ± 6.53

**Table 3:** Semi-quantitative GC–MS analysis of 3-hydroxybutanoic acid (3-HB) and 3-hydroxypentanoic acid (3-HV) methyl esters detected in biomass samples obtained from *Cupriavidus necator* cultivated on virgin wood hydrolysate (VWH), including relative peak areas and estimated monomer composition.

Sample	3-HB Peak Area	3-HV Peak Area	3-HB/3-HV Ratio	3-HB:3-HV wt%
**VWH T24**	9.21 × 10^8^	5.94 × 10^8^	1.55	61:39
**VWH T48**	1.15 × 10^9^	6.98 × 10^8^	1.65	62:38

**Table 4:** Biomass production and intracellular PHA accumulation by *Cupriavidus necator* cultivated on virgin wood hydrolysate (VWH) and detannized wood hydrolysate (DTH). Biomass concentration, PHA production, PHA yield relative to biomass (YP/X), and cultivation time corresponding to maximum PHA accumulation are reported.

Carbon Source	Biomass (g L^−1^)	PHA (mg L^−1^)	Y_P/X_ (%)	T (h)
**Virgin wood hydrolysate**	1.26 ± 0.01	68.51	5.44	48
**Detannized wood hydrolysate**	0.40 ± 0.03	0.21	6.01	120

## Data Availability

The original contributions presented in this study are included in the article. Further inquiries can be directed to the corresponding authors.

## Abbreviations

The following abbreviations are used in this manuscript:

Abbreviation	Definition
AA	Acetic acid
DTH	Detannized wood hydrolysate
DT	Detannized wood
DCW	Dry cell weight
FA	Formic acid
GAE	Gallic acid equivalents
GC–MS	Gas chromatography–mass spectrometry
HMF	5-Hydroxymethylfurfural
HPLC	High-performance liquid chromatography
IEC	Ion-exchange column
I.S.	Internal standard
LA	Levulinic acid
OD600	Optical density at 600 nm
PHA	Polyhydroxyalkanoates
PHB	Poly(3-hydroxybutyrate)
PHBV	Poly(3-hydroxybutyrate-co-3-hydroxyvalerate)
PLA	Polylactic acid
PVDF	Polyvinylidene fluoride
RFU	Relative fluorescence units
3-HB	3-Hydroxybutanoate
3-HV	3-Hydroxypentanoate
VWH	Virgin wood hydrolysate
VW	Virgin wood
YP/X	PHA yield relative to biomass
